# Effectiveness of Flapless Cortico-Alveolar Perforations Using Mechanical Drills Versus Traditional Corticotomy on the Retraction of Maxillary Canines in Class II Division 1 Malocclusion: A Three-Arm Randomized Controlled Clinical Trial

**DOI:** 10.7759/cureus.44190

**Published:** 2023-08-27

**Authors:** Doa'a Tahseen Alfailany, Mohammad Y Hajeer, Mohammad Ihsan Al-Bitar, Hallaj I. Alsino, Samer T. Jaber, Bassel Brad, Khaldoun Darwich

**Affiliations:** 1 Department of Orthodontics, Faculty of Dentistry, University of Damascus, Damascus, SYR; 2 Department of Orthodontics, Faculty of Dentistry, Al-Watanyia Private University, Hama, SYR; 3 Department of Oral and Maxillofacial Surgery, Faculty of Dentistry, University of Damascus, Damascus, SYR

**Keywords:** canine rotation, study model analysis, rate of orthodontic tooth movement, skeletal class ii division 1 malocclusion, canine retraction, cortico-alveolar perforations, traditional corticotomy, orthodontic tooth movement, orthodontic treatment, acceleration

## Abstract

Background and objectives: Both invasive and minimally invasive surgical methods have recently gained popularity in accelerating orthodontic tooth movement. Traditional corticotomy (TC) was one of the first effective invasive surgical techniques in shortening orthodontic treatment time, whereas the flapless cortico-alveolar perforations (FCAPs) technique is a modern minimally invasive method that has recently shown good results in different types of orthodontic tooth movement. Therefore, this study aimed to compare the effectiveness of TC versus FCAPs in maxillary canine retraction when treating Class II division 1 malocclusion patients.

Materials and methods: This was a single-blinded, single-center, three-arm randomized controlled trial. A total of 51 patients (22 males, 29 females, mean age 20.98 ± 1.95) whose treatment planning included the extraction of maxillary first premolars were enrolled and randomly divided into three groups: the TC group, the FCAPs group, and the control group. The assessed outcomes were the amount of canine retraction, anchorage loss, and canines’ rotation, which was evaluated at five-time points till the completion of canine retraction.

Results: There were statistically significant differences in the amount of canine retraction between the three groups in the first two months (p < 0.001), with greater mean values in the TC group (p < 0.001) in the first month. However, the amount of canine retraction in the FCAPs group was significantly greater in the second month compared to the TC group (p = 0.003) and the control group (p < 0.001). In the first month of canine retraction, anchorage loss, and canine rotation were significantly lesser in the TC and FCAPs groups than in the control group (p < 0.001). On the contrary, the canines’ rotation amount after the completion of retraction was greater in the TC group than in the other two groups (p < 0.001).

Conclusion: TC and FCAPs are efficient adjunctive surgical methods for accelerating canine retraction. At the end of the first month, the TC accelerated canine retraction by 59.85% and FCAPs by 44% compared to the conventional retraction. At the end of the second month, the acceleration was less than recorded in the first month (35.44% and 50.20%, respectively). The acceleration effect of the surgical interventions appeared transient and did not last in the following observation period.

## Introduction

The trend of using acceleration interventions to accelerate orthodontic tooth movement (OTM) has increased in recent years [1]. These interventions can be briefly classified into surgical and non-surgical [2]. The non-surgical category includes physical [3], biomechanical [4,5], and biological procedures [6]. On the other hand, surgical methods, which are the most clinically applied and tested [7], all are based on one principle, which depends on causing local and temporary damage to the alveolar bone to induce the “regional acceleratory phenomenon” (RAP) leading to accelerating the OTM [8,9]. Traditional corticotomy (TC) is one of the surgical methods that is considered the most popular, with the largest amount of research evidence proving its efficacy in accelerating OTM [10]. However, due to its invasive nature, it requires a full-thickness flap with extensive removal of the cortical bone, and therefore, patients are less willing to accept it for fear of the expected pain and swelling [11]. As a result, minimally invasive methods have recently been introduced to overcome the side effect of invasive techniques, such as piezocision-based flapless corticotomy [12-14], corticision [15], laser-assisted flapless corticotomy [16], and micro-osteoperforations (MOPs) [17].

The term “micro-osteoperforations” was first mentioned by Alikhani in 2013 as a conservative surgical intervention for the orthodontic acceleration [18]. The principle depends on creating minimally transmucosal holes within the cortical bone without flap raising. Moreover, the perforations are multiply performed near the area where the acceleration OTM is desired [19]. The MOPs or flapless cortico-alveolar perforations (FCAPs) rely on instruments such as the Propel device, a single-use device with a 1.5mm width with an adjustable depth between 3.5 and 7mm [20]. However, the relatively high price of the Propel device has prompted researchers to use other less expensive methods, such as self-drilling mini-implants to perforate the cortex and part of the superficial alveolar bone [21]. Furthermore, the drills of self-tapping mini-implants have also been utilized in some studies to create the perforations of the FCAPs [22,23] as the mechanical work is quicker [24].

 In the recent, many systematic reviews have reported the effectiveness of “FCAP” or “MOPs” on the rate of OTM [25-27]. Some previous studies have compared the efficacy of the FCAPs and piezocision in accelerating OTM. Alqadasi et al. reported that both interventions effectively accelerated the OTM without any significant difference [28]. Furthermore, Farag et al. concluded that both interventions were effective in accelerating the OTM, noting that the cortico-alveolar perforations were slightly superior to the piezocision [29].

Through a review of the published literature, no clinical study was found that evaluated the effectiveness of FCAPs using mechanical drills compared to conventional corticotomy in terms of accelerating maxillary canine retraction. Therefore, the current research aimed to evaluate the effectiveness of cortico-alveolar perforations by a mechanical drill versus TC by a piezoelectric device in retracting the upper canines in comparison with a control group in which the upper canines are traditionally retracted (without any auxiliary surgical intervention).

## Materials and methods

Study design and settings

This study was a three-arm, parallel-group randomized controlled trial (RCT) conducted at the Department of Orthodontics, Faculty of Dentistry, University of Damascus from September 2018 to August 2020. This study was registered at Clinical Trials.gov (NCT03659188). The Local Research Ethics Committee Approval was obtained (UDDS-550-01042019/SRC-3310) and was funded by the University of Damascus Postgraduate Research Budget (Ref no: 80253000112DEN).

Sample size estimation

The sample size was calculated using the Minitab® 18.1 software (Minitab LLC, Pennsylvania, USA) with an alpha level of 0.05 and a power of 80%. The variable of interest in the current study was the acceleration of the upper canines' retraction. According to Al-Naoum et al., the standard deviation for this variable was 0.33 [9]; the least clinically significant difference to be detected was a change of 0.38 mm/month. Using a one-way ANOVA, the required sample size was 16 patients for each group. However, one patient was added to each group to prevent possible withdrawal, bringing the total number required to 51 patients (i.e., 17 patients in each group).

Patients' recruitment and inclusion in the study

**Figure 1:**
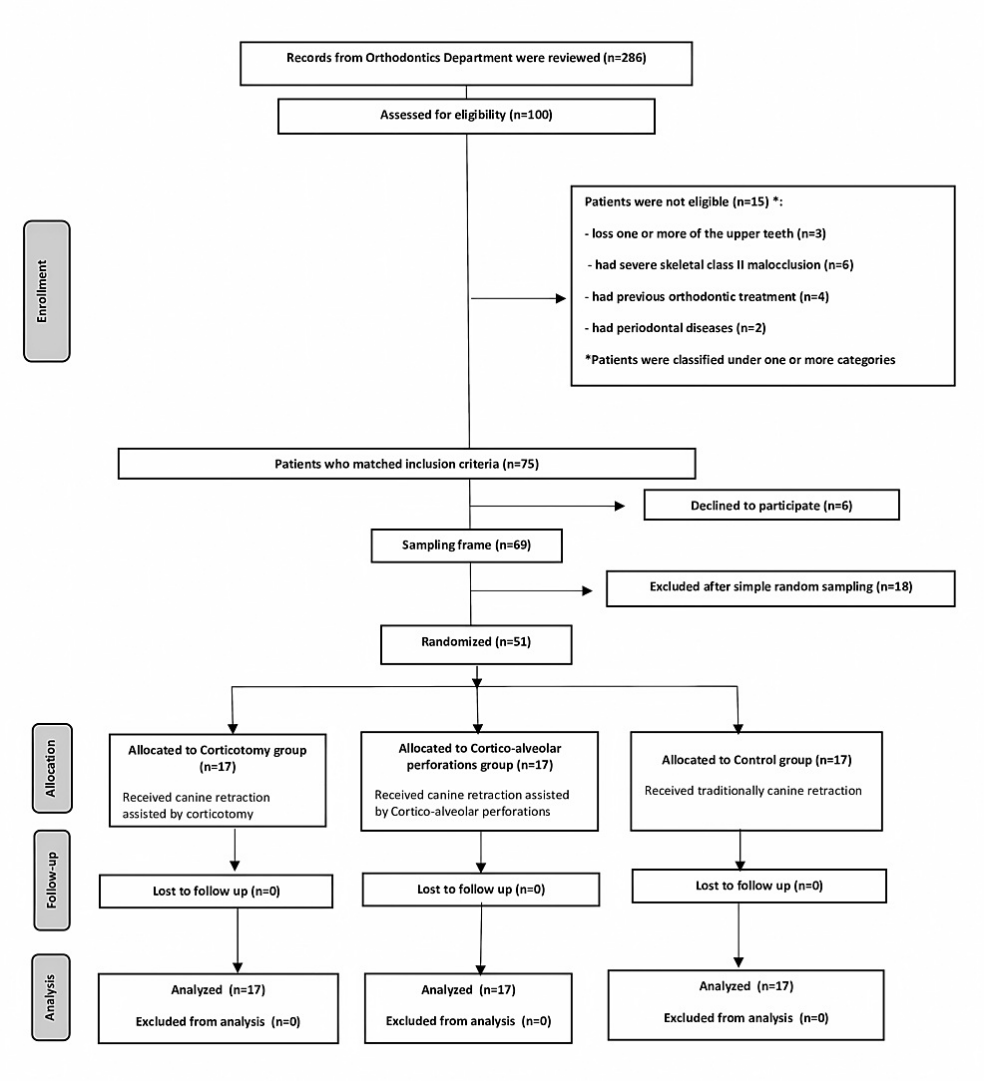
The Consolidated Standards of Reporting Trials (CONSORT) flow diagram of patient recruitment, follow-up, and entry into data analysis.

After a clinical assessment of 100 patients at the Department of Orthodontics at the Faculty of Dentistry at Damascus University, it was found that 75 patients met the inclusion criteria. Later, six patients refused to participate in the study after providing enough information about this research project. Subsequently, 51 of the 69 candidate patients were randomly selected and divided into three treatment groups (Figure 1). After the presentation of the information sheet to the patients, informed consent was obtained.

All patients fulfilled the following inclusion criteria: (1) age range of 18-28 years (2) skeletal class II malocclusion (skeletal relationship in the sagittal plane (ANB)=5-9); (3) mild to moderate crowding of upper anterior teeth (2-6 mm); (4) normal or vertical growth pattern (which was assessed radiographically); (5) 5- to 10-mm of overjet and 1- to 4-mm of the overbite; (6) the presence of a complete upper permanent dentition (except third molars). The exclusion criteria involved the following: (1) patients with contraindications to anesthesia and surgical intervention; (2) previous orthodontic treatment; (3) poor oral hygiene; (4) presence of periodontal diseases, which was assessed clinically (probing depth > 4 mm, plaque and gingival index >1 [30]).

Randomization, allocation concealment, and blinding

To assign the 51 patients to three treatment groups, Minitab® Version 19.1 (Minitab Inc., Pennsylvania, USA) produced a list of random numbers with a 1:1:1 allocation ratio, i.e., 17 participants in each treatment group. Opaque, numbered, and locked envelopes were used to hide the allocation sequence until the end of the leveling and alignment phase when it was opened. The random allocation sequence was conducted by an academic specialist who was not participatory in this study. Because of the nature of the interventions, blinding neither the practitioner nor the patients was possible. Therefore, blinding was limited only to the outcomes' assessor.

Orthodontic procedures

One week before the commencement of orthodontic treatment, the first upper premolars were extracted at the Department of Oral and Maxillofacial Surgery for all patients. Subsequently, the fixed orthodontic appliance (McLaughlin, Bennett, and Trevisi (MBT) system) 0.022-inch bracket-slot prescription (Votion™, Ortho Technology®, FL, USA) was applied to the three treatment groups. Then, the sequencing of wires to the end of leveling and alignment was started, i.e., until applying a 0.019 × 0.025-in stainless steel wire, which was considered the basal archwire. However, to achieve moderate anchorage, soldered transpalatal arches were placed from the treatment beginning.

Surgical intervention

Before the commencement of the surgical procedures, all patients were demanded to rinse with 0.12% chlorhexidine gluconate for a minute. After that, local anesthesia was administered to mesial and distal upper canine at both sides by supra-periosteal buccal infiltration and sub-periosteal palatal one (Lidocaine HCL 2% - Epinephrine 1:80,000). After finishing the surgical interventions, the surgical sites were covered by pieces of iodoform gauze. The patient was instructed post-operative regimen, which included taking two tablets of antibiotic per day for one week after corticotomy, applying ice packs for the first 12 hours after corticotomy, eating soft food in the first three days, using a mouthwash during the first few days, and avoiding smoking during the first week. Instead of taking non-steroid anti-inflammatory drugs, which were not permitted to avoid overlapping with the RAP effect, only 500 mg acetaminophen (Panadol) was allowed to be taken when necessary [31,32].

TC group (TCG)

**Figure 2:**
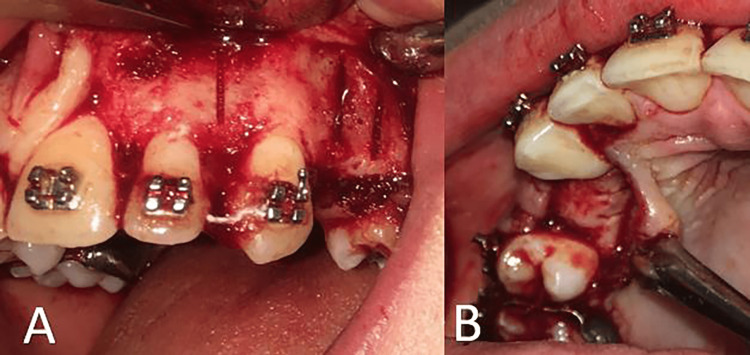
Performing the conventional corticotomy: (A) the buccal side; (B) the palatal side.

The TC was conducted under the supervision of one of the coauthors (B.B.) at the Oral and Maxillofacial Surgery Department, Damascus University. After applying the local anesthesia and for the sake of exposing the cortical bone, a full-thickness flap was elevated buccally and palatally on the maxillary canines at both sides using a surgical scalpel blade no. 15. The buccal flap extended from the mesial of the lateral incisor to the distal of the upper second premolar. At the same time, the palatal flap extended from the mesial of the canine to the distal of the upper second premolar. This was followed by performing two vertical cortical cuts with 3mm depth from the buccal and palatal aspects at the extraction site using a piezoelectric knife (BS1). Moreover, a vertical cortical incision was also made on the canine from the buccal aspect (Figures 2A, 2B) mesially. The corticotomy cuts started 2-3 mm below the alveolar crest to protect the crystal bone. Before repositioning and suturing the flaps, physiological serum was used to wash the area and remove the osteoporosis and blood clots. The suturing was done with the interrupted technique using a nonabsorbent 3-0 silk. One week after the corticotomy, sutures were removed.

FCAPs group (FCAPG)

**Figure 3:**
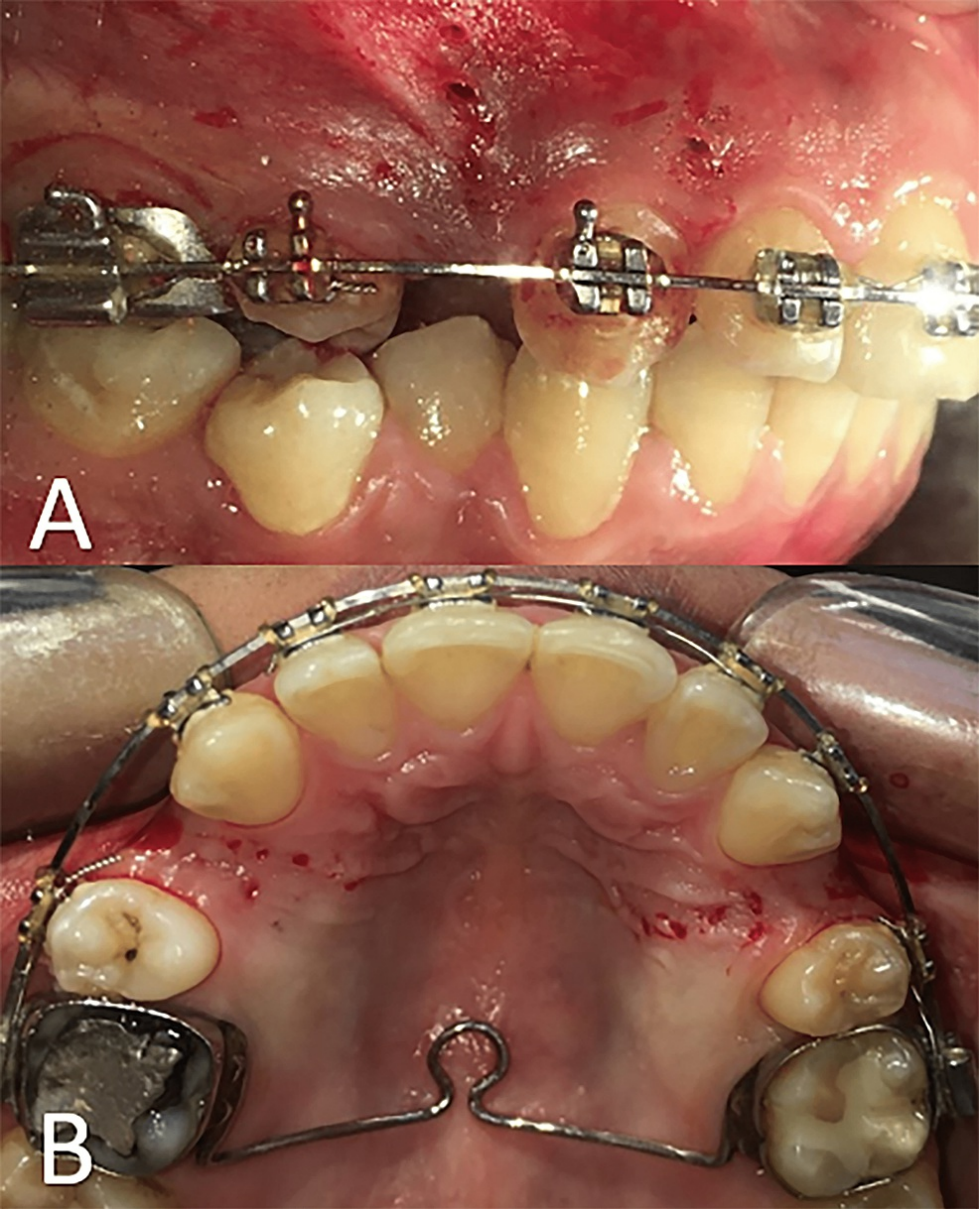
Performing the flapless cortico-alveolar perforations: (A) the buccal side; (B) the palatal side.

Mini-implant drills designed to create an entrance for mini-implants (Aarhus, American Orthodontics, Sheboygan, WI, USA) with dimensions of 0.9 × 4 × 22 mm were used on a contra-angle handpiece with irrigation at a slow speed. However, five holes from the buccal aspect were made by the drill distal to the upper canine on each side, and five corresponding holes were made from the palatal side. Moreover, one perforation was also performed mesial to the canine from the buccal aspect. Twelve buccal and ten palatal perforations were created on both sides (Figures 3A, 3B). These holes were 1.5-2 mm apart in the area between the canine and the second premolar, and the two mesial holes were 4 mm away from the gingival papilla in the middle of the distance between the canine and the lateral incisor. Each perforation was made with a width of 0.9 mm and a depth of 4 mm, which was measured and controlled continuously during surgery using a UNC 15 probe.

Orthodontic procedure: canine retraction

The canine retraction was initiated after three days post-operation. For all patients, 0.019 × 0.025-inch stainless steel archwires with nickel-titanium closed-coil springs (NT3 closed coil, American Orthodontics, Sheboygan, Wis, USA) stretched from canine brackets hooks' to first molars bands that applied 150 grams on each side were used to retract the canines. The generated force was measured using a force gauge (040-711-00; Dentaurum, Ispringen, Germany). The follow-up appointments for patients were biweekly to achieve the maximum advantages of RAP [16]. The force generated was verified and adjusted to 150 grams at each follow-up appointment if necessary.

Outcomes measures: dental cast analysis

**Figure 4:**
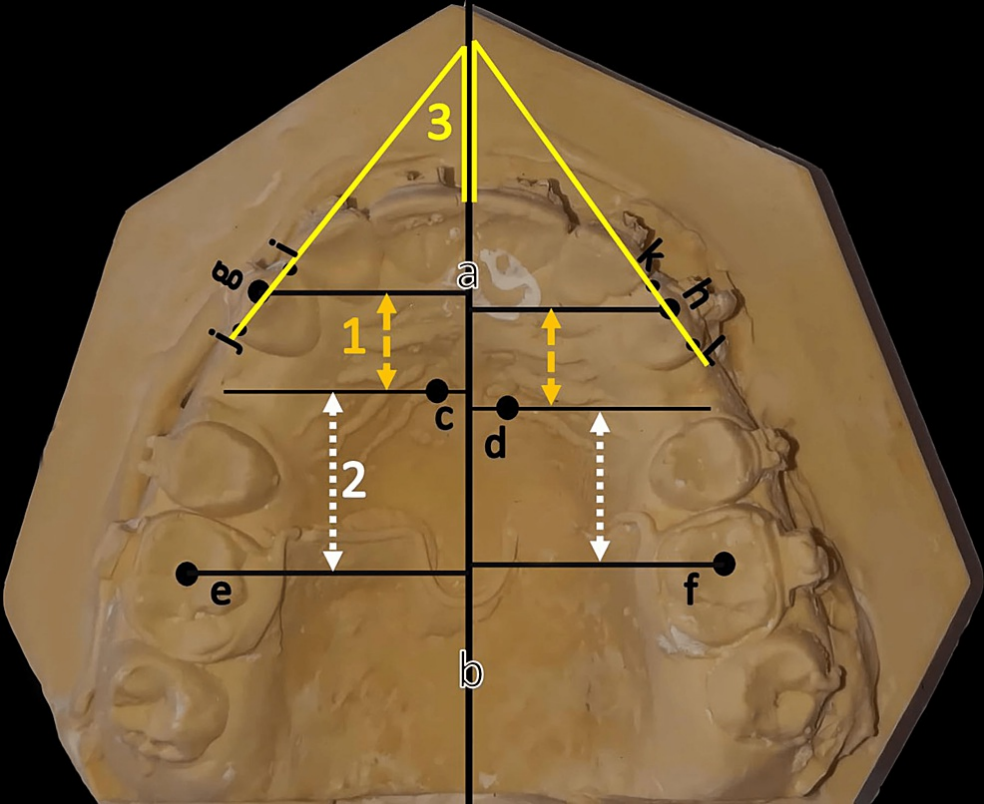
Reference points and measurements made on the dental cast (a,b) Mid-palatal suture line (i.e., the reference plane), (c) medial end of right third palatal ruga, (d) medial end of left third palatal ruga, (e) central fossa of upper right first molar, (f) central fossa of upper left first molar, (g) cusp tip of the right maxillary canine, (h) cusp tip of the left maxillary canine, (i) mesial margin of the maxillary right canine, (j) distal margin of the right maxillary canine, (i) mesial margin of the maxillary left canine, and (j) distal margin of the left maxillary canine.

Dental casts were obtained at five-time points: before the onset of canine retraction (T0), after one month (T1), after two months (T2), after three months (T3), and at the end of the canine retraction phase (T4). However, the amount of canine retraction was assessed as a primary outcome measure, whereas the secondary outcome measures were the loss of molar anchorage and canines’ rotation. Initially, reference points were determined on the casts based on the method described by Ziegler and Ingervall [33]. However, reference points and their definitions are given in Figure 4. After that, the measurements of the following variables were conducted: (1) the distance between the medial ending of the third palatal rugae and the tip of the upper canine cusp to assess the anterioposterior of the canine movement, (2) the distance between the medial ending of third palatal rugae and the central fossa of upper first molar to assess the anterioposterior molar movement, and (3) the angle between the mid-palatal suture and the line through the mesial and distal edges of the maxillary canine to assess canine rotation. However, the measurements were calculated manually by the researcher (M.B.) using a digital caliper (REF 042-751-00, Dentaurum, Ispringen, Germany).

Statistical analysis

Two packages of statistical programs were used to analyze the data: Minitab® (Version 19.1; Minitab Inc.) and SPSS® (version 24.0; IBM Corp., Armonk, NY, USA). The normality of data distributions was evaluated using the Anderson-Darling Normality tests. A one-way ANOVA test was used to compare the dental casts variables between three evaluated groups. For pairwise comparisons, the Bonferroni post-hoc test was applied. The results were deemed significant when p<0.05, except for the one-way ANOVA test, which was adjusted according to Bonferroni's correction.

The reliability of the used method

After one month interval, twenty dental casts were randomly selected and remeasured by the same assessor (M.A.). The interclass correlation coefficients were applied to check the intra-examiner reliability (random error), while paired-sample t-tests were conducted to detect any systematic errors.

## Results

Baseline sample characteristics

Fifty-one patients (22 males, 29 females; mean ages ± standard deviation (SD): 20.98 ± 1.95 years) were enrolled in this study. The basic characteristics of the included patients are displayed in Table 1. No dropout occurred; therefore, the statistical analysis included all the patients.

**Table 1:** Basic sample characteristics (gender and age). * Employing one-way ANOVA, †employing chi-square test n: number of patients, SD: standard deviation, TC:  Traditional corticotomy, FCAPs:  Flapless cortico-alveolar perforations.

Group	Gender n (%)	P-value^*^	Mean Age (SD)	P-value†
TC (n=17)	Male 6 (35.29%)	0.571	21.23 ± 2.33	0.647
	Female 11 (64.7%)			
FCAPs (n=17)	Male 9 (52.94%)		21.09 ± 2.06	
	Female 8 (47.05%)			
Contol (n=17)	Male 7 (41.17%)		20.62 ± 1.48	
	Female 10 (58.82%)			
All sample (n=51)	Male 22 (43.13%)		20.98 ± 1.95	
	Female 29 (56.86%)			

The error in the method

The interclass correlation coefficients for the measures manifested strong intra-examiner reliability, ranging from 0.982 to 0.999, indicating high intra-examiner reliability. Moreover, paired sample t-test for the two measurements showed no significant difference (p > 0.05), indicating no systematic errors.

Dental cast analysis

For the side disparities in the amount of canine retraction, molar mesial movement, and canine rotation, means were calculated individually for the right and left sides. However, no significant differences were found between the right and left sides in the three studied groups. Therefore, the means of both sides were used to calculate the amount of canine retraction, anchorage loss, and canine rotation.

The mean distance traveled by the upper canines was significantly greater in the surgical intervention groups than in the control group during the first two months of the retraction stage (p < 0.001; Table 2). On the other hand, no statistically significant differences were found after three months and at the end of canine retraction between the three studied groups (p = 0.029, p = 0.478). However, after a month of retraction commencement, the TC group showed the greatest amount of canine retraction (mean value of 2.79 ± 0.39 mm) with a statistically significant difference when compared with the other two groups (p < 0.001, Table 3). On the other hand, the canines' retraction amount in the second month was significantly greater in the FCAPs group than in the TC group (p = 0.003) and the control one (p < 0.001). Regarding the loss of molar anchorage, the least amount of anchorage loss was observed after the first month of canine retraction in the TC group (mean value of 0.57 ± 0.24 mm), while the control group showed the highest amount of anchorage loss (mean value of 2.09 ± 0.79 mm) with a significant difference in comparison with the two surgical intervention groups (p < 0.001). On the contrary, the TC group, after three months, showed a significantly greater amount of anchorage loss (mean value of 1.93 ± 0.68 mm) when compared with the FCAPs group and the control one (p = 0.001, p < 0.001, respectively).

**Table 2:** Descriptive statistics of the observed changes in the dental casts variables between assessment times in the three groups and the results of the significance test† † Employing One-way ANOVA between the three groups at each time interval with an adjusted alpha level due to the repeated assessments using Bonferroni's correction (0.05/4 = 0.012), * statistically significant at p < 0.012 n: number of patients, CI: confidence interval, SD: standard deviation, T0: before the onset of canine retraction, T1: after one month, T2: after two months, T3: after three months, T4: at the end of the canine retraction phase

Variables	Time	Traditional corticotomy (n=17)			Flapless cortico-alveolar perforations (n=17)			Control (n=17)			P-value
		Mean (SD)	95%CI of the mean		Mean (SD)	95%CI of the mean		Mean (SD)	95%CI of the mean		
			Lower	Upper		Lower	Upper		Lower	Upper	
Amount of canine retraction (in mm)	T0-T1 (1^st ^month)	2.79 (0.39)	2.60	2.99	2.00 (0.35)	1.82	2.19	1.12 (0.16)	1.04	1.20	<0.001*
	T1-T2 (2^nd^ month)	1.89 (0.42)	1.68	2.11	2.45 (0.59)	2.15	2.75	1.22 (0.32)	1.06	1.38	<0.001*
	T2-T3 (3^rd^ month)	1.05 (0.40)	0.85	1.26	0.86 (0.32)	0.70	1.03	1.19 (0.31)	1.03	1.35	0.029
	T3-T4 (The end of canine retraction)	1.08 (0.44)	0.17	2.33	1.68 (0.59)	1.38	1.99	1.52 (0.58)	1.22	1.81	0.478
Anchorage loss (in mm)	T0-T1 (1^st ^month)	0.57 (0.24)	0.45	0.69	0.76 (0.33)	0.60	0.94	2.09 (0.79)	1.68	2.50	<0.001*
	T1-T2 (2^nd^ month)	0.90 (0.53)	0.62	1.17	0.93 (0.58)	0.63	1.23	0.94 (0.60)	0.63	1.24	0.975
	T2-T3 (3^rd^ month)	1.93 (0.68)	1.58	2.28	1.08 (0.75)	0.70	1.47	0.79 (0.53)	0.52	1.06	<0.001*
	T3-T4 (The end of canine retraction)	0.60 (0.61)	0.29	0.92	0.34 (0.42)	0.13	0.56	0.59 (0.30)	0.43	0.74	0.202
Canine rotation (in degrees)	T0-T1 (1^st ^month)	4.24 (2.99)	2.70	5.78	8.89 (2.67)	7.52	10.27	12.95 (3.47)	11.17	14.74	<0.001*
	T1-T2 (2^nd^ month)	7.02 (4.43)	4.74	9.30	3.70 (1.67)	2.84	4.56	5.30 (1.78)	4.38	6.22	0.007*
	T2-T3 (3^rd^ month)	2.40 (2.37)	1.18	3.63	4.02 (1.16)	3.43	4.62	3.29 (1.90)	2.28	4.30	0.054
	T3-T4 (The end of canine retraction)	5.76 (3.11)	4.16	7.37	2.73 (1.62)	1.90	3.57	1.94 (0.91)	1.47	2.41	<0.001*

**Table 3:** Post-hoc tests for canine retraction amount, anchorage loss, and canine rotation in pairwise comparisons. † † Employing Bonferroni's post-hoc tests, *: statistically significant at p < 0.05 T0-T1: Changes that occurred in the first month, T1-T2: Changes that occurred in the second month, T2-T3: Changes that occurred in the third month, T3-T4: at the end of canine retraction phase, TCG: traditional corticotomy group, FCAPG: flapless cortico-alveolar perforations group, CG: control group

Variable	Time	Comparison groups	Mean difference	P-value
Canine retraction amount	T0-T1	TCG vs. FCAPG	0.79	<0.001*
		TCG vs. CG	1.67	<0.001*
		FCAPG vs. CG	0.88	<0.001*
	T1-T2	TCG vs. FCAPG	-0.56	0.003*
		TCG vs. CG	0.67	<0.001*
		FCAPG vs. CG	1.23	<0.001*
Anchorage loss	T0-T1	TCG vs. FCAPG	-0.19	0.806
		TCG vs. CG	-1.52	<0.001*
		FCAPG vs. CG	-1.33	<0.001*
	T2-T3	TCG vs. FCAPG	0.85	0.001*
		TCG vs. CG	1.14	<0.001*
		FCAPG vs. CG	0.29	0.587
Canine rotation	T0-T1	TCG vs. FCAPG	-4.65	<0.001*
		TCG vs. CG	-8.71	<0.001*
		FCAPG vs. CG	-4.06	0.001*
	T3-T4	TCG vs. FCAPG	3.03	<0.001*
		TCG vs. CG	3.82	<0.001*
		FCAPG vs. CG	0.79	0.823

Regarding the retracted canines' rotation, there were statistically significant differences between the three treatment groups in the first and second months and after the end of retraction (p < 0.001, p = 0.001, p < 0.001, respectively). After completion of retraction, the amount of canines’ rotation was significantly greater in the TC group when compared with the FCAPs group and the control one (p < 0.001), while no significant difference was detected between the FCAPs group and the control group in this regard (p = 0.823).

Harms

No harm was observed during treatment in TC or cortico-alveolar perforations groups.

## Discussion

According to our knowledge, no single trial has been carried out to compare one of the most invasive techniques (TC) with one of the recent minimally invasive techniques (FCAPs) regarding canine retraction acceleration. Therefore, this is the first clinical 3-arm, parallel-group RCT.

To eliminate the confounding effect of extraction on the RAP following the application of surgical interventions [10], the maxillary first premolars were extracted at the beginning of orthodontic treatment, i.e., before the leveling and alignment phase, which took three to four months before the commencement of the maxillary canine's retraction at both sides. Closed NiTi coil springs were used to distalize the upper canines at both sides instead of elastomeric chains due to their continuous light force generation and the possibility of establishing better oral health [12]. A bilateral canine retraction was initiated after three days of surgical interventions to take advantage of the RAP [10].

The amount of canine retraction was significantly greater in the surgical intervention groups than in the control one during only the first two months of retraction commencement. Thereafter, no significant differences were found between the three groups. This may be attributed to the transient effect of “RAP,” which begins within days of the surgical injury, reaches its peak within the first and second month, and then gradually disappears over time [7]. However, the previous finding aligned with the study of Sharma et al., who found that corticotomy speeds the canine retraction rate in the following two months post-corticotomy [34]. In contrast, we disagreed with the results of previous studies [35,36], which indicated that FCAPs were ineffective in accelerating upper canine retraction. This may be attributed to their application of only three cortical holes in the buccal aspect, whereas in our study, 11 holes were applied both in the buccal and palatal regions on each side. TC accelerated the rate of maxillary canine distalization by 59.85% after one month, while the acceleration rate in the second month was 35.44% compared to the control group. These findings agree with Abbas et al. [37], who reported that corticotomy exhibited 1.5 to two times faster canine retraction than traditional retraction. On the other hand, FACPs accelerated the rate of canine retraction during the first two months (by 87.5% and two times, respectively) compared with the control group. This was in line with the systematic review of Mohaghegh et al., who found that FCAPs increase the rate of OTM in the canine retraction-based treatment [38]. The TC group showed greater canine movement in the first month than the FCAPs group. This may be because corticotomy requires extensive surgical intervention, which may have enhanced the RAP to a greater extent [37].

The current study showed that the amount of molar anchorage loss after one month of retraction was significantly greater in the control group compared to the surgical intervention groups. This can be explained by the fact that the reaction of the alveolar bone toward performing the TC and FCAPs was localized in the place of surgical intervention without extending to the posterior segment [2]. Thus, their effect on the first molar decreased, and the anchorage loss was slower than the conventional retraction.

At the end of canine retraction, the canines’ rotation in both surgical groups was greater than the control one. This can be caused by increased canine distalization in the surgical groups and decreased bone density due to surgical procedures. However, the increase in the canine rotation was significantly greater in the TC group than in the FCAPs and the control groups. This may be explained by the invasive nature of the TC, creating a significant weakening of the cortical-alveolar bone that would have allowed the maxillary canines to rotate significantly during retraction.

Limitations

In the present study, the application of acceleration procedures was limited to the upper jaw, and the effectiveness of these procedures in the acceleration of lower canine retraction was not evaluated. Furthermore, this trial did not evaluate patient-centered outcomes, skeletal outcomes, and the potential effect of gender on the rate of OTM. Additionally, future research should consider the possible associated harms, such as root resorption, scars, periodontal status, and tooth vitality.

Generalizability

The current RCT was a single-center trial on a specified type of malocclusion (class II division 1) within a specified age range (18-27 years) according to strict selection criteria. Therefore, the findings of the present trial can only be generalized to patients who undergo orthodontic treatment similar to that in this study.

## Conclusions

Both TC and FCAPs are effective procedures in accelerating the retraction of the upper canines compared to the control group. At the end of the first month, the TC accelerated canine retraction by 59.85% and FCAPs by 44% compared to the conventional retraction. At the end of the second month, the acceleration was by (35.44%, and 50.20%; respectively). Compared to the control group, both surgical techniques showed less anchorage loss and canine rotation at the end of the first month of retraction commencement.
